# Malignant Nodular Hidradenoma of the Eyelid: A Rare Sweat Gland Tumor

**DOI:** 10.4103/0974-9233.71595

**Published:** 2010

**Authors:** Abdulla Al Baghli, Suresh S. Reddy, Maragaret A. Reddy

**Affiliations:** Department Oculoplastic Surgery, Al-Bahar Eye Centre, Ibn Sina Hospital, Safat, Kuwait

**Keywords:** Eccrine Glands, Nodular Hidradenoma, Sweat Gland Tumor

## Abstract

We report a case of malignant nodular hidradenoma in a middle-aged man, who presented with a nodular swelling in the eyelid. The tumor was similar to its benign counterpart but had additional features such as surface ulceration, numerous mitiotic figures, and an infiltrative growth pattern. Malignant forms of hidradenomas are unusual and the possibility this variant should be considered in the differential diagnosis of eyelid tumors.

## INTRODUCTION

Nodular hidradenoma is a cutaneous neoplasm that can occur at various sites. It has been referred to as clear cell hidradenoma or eccrine sweat gland adenoma.^1^ Hidradenomas arise as intradermal nodules from eccrine sweat glands. Ultrastructural and enzyme histochemical studies have shown nodular hidradenomas to be intermediate between eccrine poroma and eccrine spiradenoma.^2^ The histology of the malignant hidradenoma resembles that of its benign counterpart. The criteria for malignancy include poor circumscription, presence of nuclear atypia, mitotic activity, presence of predominantly solid cell islands, infiltrative growth pattern, necrosis, and angio-lymphatic permeation.^3^–^5^ Sweat gland tumors of the eyelid are extremely rare yet the possibility of sweat gland tumors should be considered during differential diagnosis of eyelid tumors. The malignant forms are distinctly unusual. We report a case of malignant nodular hidradenoma in a middle-aged man, who presented with a nodular swelling in the right upper eyelid.

## CASE REPORT

**Figure 1:**
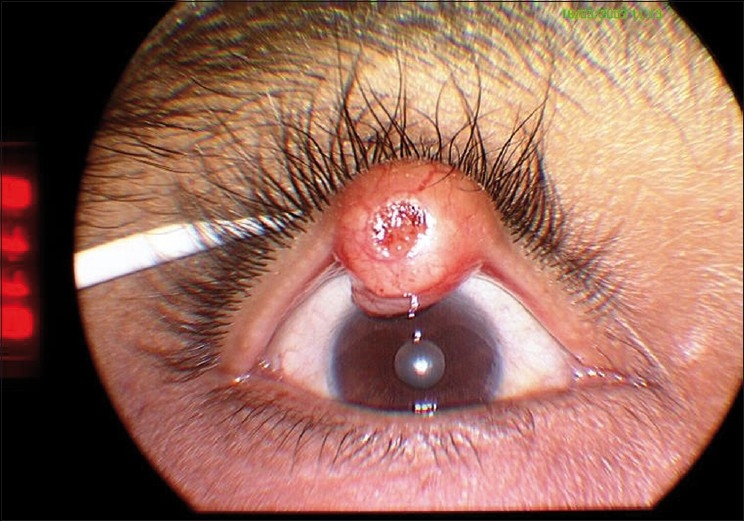
Clinical appearance of the lesion on the right upper lid

A 51-year-old man presented to our institute with an enlarging, painless, nodular mass in the right upper lid that began 4 months prior with rapid increase in size over the last 3 months. The mass became significantly prominent over the last month prior to presentation resulting in discomfort, ulceration, and bleeding on manipulation. Physical examination revealed a solitary mass in the middle third of the right upper eyelid, overhanging the lid margin that was 9 mm × 12 mm in size, pinkish, firm, fleshy with small-dilated blood vessels on the surface with central ulceration and crusting [Figure 1]. The mass was underlying the right upper lid skin extending beyond the lash line without invading the palpebral conjunctiva. It was firm to hard in consistency and tender to the touch and did not appear to extend to the deeper underlying tissues. There was no regional aurical, cervical, or submandibular lymphadenopathy. The remainder of the ocular and general physical examination including examinations of the liver and lungs was normal. Basal cell carcinoma or squamous cell carcinoma was suspected on the basis of the clinical examination. A biopsy was sent to the pathology service.

**Figure 2:**
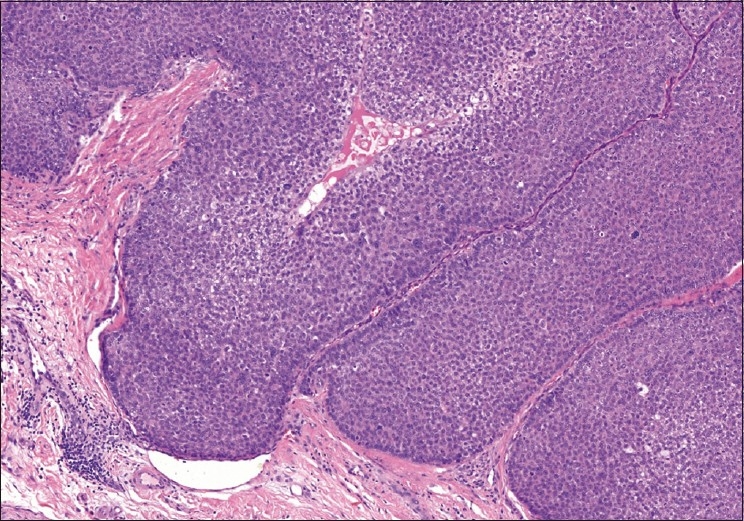
Tumor cells within hyalinized stroma (H and E, ×200)

A complete, wide excision of the nodular mass with a 3 mm clear margin of healthy surrounding tissue was performed along with primary closure [Figure 2]. The pathology service identified malignant nodular hidradenoma, a rare eccrine sweat gland tumor (described below). At the last visit, 6 months after excision, there was no recurrence of the lesion. The patient was subsequently lost to follow up.

### Pathologic findings

#### Gross description

The specimen submitted to the pathology department comprised of a nodular mass 1.5 cm in diameter. The cut surface was yellowish with few cavities.

#### Microscopic description

**Figure 3:**
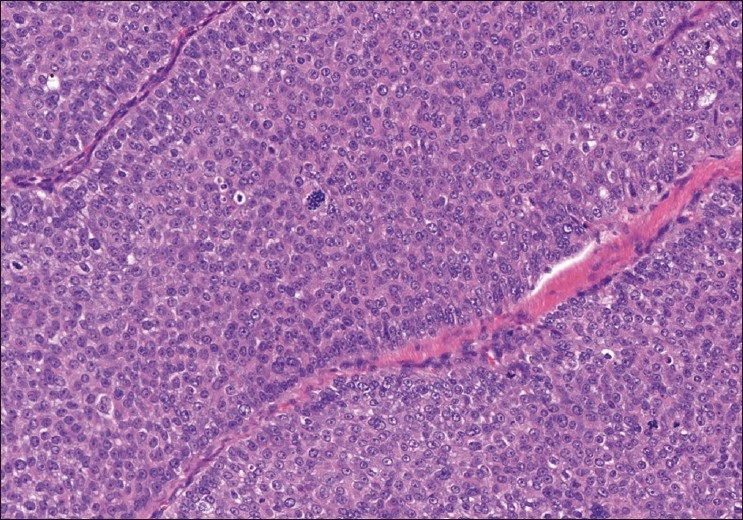
Tumor cells with predominant polyhedral cell-type and few clear cell-type cells (H and E, × 400)

**Figure 4:**
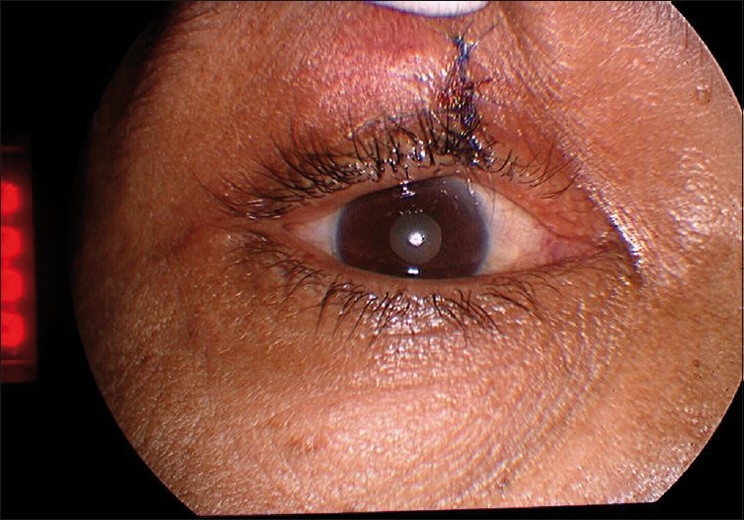
Clinical appearance after excision of the tumor mass

Histopathologic examination revealed a tumor in the dermis with surrounding fibrous capsule. Two types of tumor cells were noted with predominance of one cell-type which was polyhedral to round in configuration with round to oval nuclei and prominent nucleoli surrounded by faintly basophilic cytoplasm. The second cell-type was clear, round to oval with eccentric nucleus. The tumor cells were arranged in lobules and separated by delicate fibrous collagenous tissue [Figure 3]. The lobules were lined by cuboidal or columnar cells with few cystic spaces, brisk mitotic figures with surface ulceration, and infiltrating margins. No areas of necrosis were noticed. The tumor cells exhibited an intracytoplasmic substance that stained positively with periodic acid-Schiff stain and were diastase-sensitive. There was positive immunohistochemical reactivity to epithelial membrane antigen. A diagnosis of malignant nodular hidradenoma was performed on the basis of the histopathologic and immunohistochemical studies [Figure 4].

## DISCUSSION

Tumors arising from the sweat glands of the eyelids are uncommon, and the differences in their structure have given rise to much confusion. A wide variety of descriptive terms have been applied to sweat gland tumors.^6^

Histologically, sweat glands may be either eccrine or apocrine in nature. Eccrine glands are present throughout the skin but are most abundant in the palms, soles, and axillae. In the eyelid, eccrine glands are present at the lid margin and in the surface dermis. Apocrine glands are found in relatively fewer regions of the body, mainly the axillae, around the nipples, the anogenital region, and occasionally a small number on the abdomen and chest.^7^

Eccrine hidradenomas are also referred as nodular hidradenoma or clear cell hidradenomas arise from the eccrine sweat glands. This nomenclature is based on electron microscopy and histochemical studies.^8^ Clinically, hidradenoma usually presents as a solitary intradermal nodule which is well circumscribed and may appear encapsulated.^1^ However, hidradenomas do not present with discrete clinical characteristics that allows differentiation from other lid tumors on the basis of the clinical and macroscopic findings. Thus, clinical diagnosis is difficult at best and we believe any lesion which shows evidence of enlargement should be warrants histological evaluation.^9^

The differential diagnosis includes primary skin tumors with follicular, sebaceous, or sweat gland differentiation. Hidradenomas can mimic cutaneous metastatic disease from clear cell tumors such as renal cell carcinoma.^5^ The possibility of a primary basal cell carcinoma with eccentric differentiation and a lobular, hyalinised syringoma should also be included in the histological differential diagnosis.^7^ Histopathologically, hidradenoma is composed of epithelial lobules within the dermis, showing tubular lumina lined by cuboidal or columunar cells with cystic spaces that contain faintly eosinophilic material. The solid component of the tumor is comprised of two types of cells. One cell-type is round to polyhedral in shape with rounded nuclei and distinct nucleoli with slightly basophilic cytoplasm surrounding it. The second cell-type is a glycogen-rich clear cell with distinct boundaries and a small eccentric nucleus. Some tumors are composed almost entirely of clear cells hence the name clear cell hidradenomas.^8^ Tumor nodules are also associated with foci of characteristic hyalinized stroma.^10^ In our patient clear cells were not predominant, hence we classified the lesion as a nodular hidradenoma.^11^

The hidradenoma in our case had similarities to its benign counterpart but exhibited ulceration, brisk mitotic figures, and an infiltrative growth pattern suggestive of a malignant lesion. Malignant hidradenomas are most often malignant from inception as seen in our case. Some malignant hidradenomas develop from their benign counterparts. These tumors in contrast to the benign forms, tend to invade the surrounding tissue and have a high incidence of recurrence and distant metastasis.^9^

Malignant hidradenoma are distinctly unusual and are extremely rare in the eyelids. The possibility of sweat gland tumors should be considered in the differential diagnosis of eyelid tumors to ensure early detection. Management involves complete excision of the lesion.
